# Numerical investigation of the effect of cannula placement on thrombosis

**DOI:** 10.1186/1742-4682-10-35

**Published:** 2013-05-16

**Authors:** ChiWei Ong, Socrates Dokos, BeeTing Chan, Einly Lim, Amr Al Abed, Noor Azuan Bin Abu Osman, Suhaini Kadiman, Nigel H Lovell

**Affiliations:** 1 Faculty of Biomedical Engineering, University of Malaya, Malaysia University of Malaya, 50603 Kuala Lumpur, Malaysia; 2 Graduate School of Biomedical Engineering, University of New South Wales, Sydney 2052 Australia; 3 Department of Anaesthesiology, National Heart Institute, 145 JalanTunRazak, 50400 Kuala Lumpur, Malaysia

**Keywords:** Heart, LVAD, Cannula, Fluid structure interaction, Vortex, Thrombus

## Abstract

Despite the rapid advancement of left ventricular assist devices (LVADs), adverse events leading to deaths have been frequently reported in patients implanted with LVADs, including bleeding, infection, thromboembolism, neurological dysfunction and hemolysis.

Cannulation forms an important component with regards to thrombus formation in assisted patients by varying the intraventricular flow distribution in the left ventricle (LV). To investigate the correlation between LVAD cannula placement and potential for thrombus formation, detailed analysis of the intraventricular flow field was carried out in the present study using a two way fluid structure interaction (FSI), axisymmetric model of a passive LV incorporating an inflow cannula. Three different cannula placements were simulated, with device insertion near the LV apex, penetrating one-fourth and mid-way into the LV long axis. The risk of thrombus formation is assessed by analyzing the intraventricular vorticity distribution and its associated vortex intensity, amount of stagnation flow in the ventricle as well as the level of wall shear stress. Our results show that the one-fourth placement of the cannula into the LV achieves the best performance in reducing the risk of thrombus formation. Compared to cannula placement near the apex, higher vortex intensity is achieved at the one-fourth placement, thus increasing wash out of platelets at the ventricular wall. One-fourth LV penetration produced negligible stagnation flow region near the apical wall region, helping to reduce platelet deposition on the surface of the cannula and the ventricular wall.

## Introduction

Cardiovascular disease is the number one cause of death on a global scale according to the 2013 WHO report [1]. Congestive heart failure (CHF), major class of cardiovascular disease, is a serious health condition characterized by the inability of the heart to supply adequate blood flow and therefore oxygen delivery to the body. Due to limited availability of donor organs and limitations in drug therapies, left ventricular assist devices (LVADs) serve as a promising therapy for restoration of blood flow. With the advancement of technology, LVADs have gradually moved from bridge-to-transplant to destination therapy.

An LVAD is intended to restore heart function by partially or fully taking over the pumping function of the left ventricle (LV) without removing the heart from the body. It consists of a blood pump connected to the patient’s circulation with cannulae or grafts. The use of an LVAD helps to extend the life of the patient while waiting for a donor heart, that is, as a bridge to transplant. Despite their various advantages, adverse events leading to death have been frequently reported in patients implanted with LVADs including bleeding, infection, thromboembolism, neurological dysfunction and hemolysis [2-4]. One major complication associated with LVADs is device-related thrombus formation, caused by activation of coagulation in the presence of a foreign surface, as well as non-physiological flow field environments in the LV [5-7]. Hund [8] has identified three main flow conditions which trigger thrombosis or platelet activation, i.e. high shear stress, stagnation zones and recirculation zones. Regions of stagnation and recirculation (long residence time) promote thrombosis through the accumulation of agonists and active platelets. On the other hand, high shear stress triggers thrombosis by directly activating the platelets, as well as indirectly by increasing the rate of hemolysis which then induces platelet activation [8,9].

In order to optimize how the LVAD unloads the LV and to reduce thrombosis, a detailed understanding of the impact of the device on LV hemodynamic variables, such as flow dynamics and cardiac wall motion is crucial. However, detailed intraventricular flow fields in the LV cannot be directly observed through standard imaging modalities, e.g. magnetic resonance imaging (MRI) cannot be utilized due to metallic components in the pump [10]. Advanced ultrasound techniques such as the transthoracic echocardiography, have often been used to compensate for the insufficiency of standard imaging modalities. However, it remains difficult to detect the presence of thrombi using ultrasound as a thrombus is indistinguishable from fully stagnated blood in the LV [11]. As a result, *in vivo* and *in vitro* experiments (mostly using the particle image velocimetry method (PIV) [12]), as well as numerical models have been widely used.

The design of the inflow cannulae as well as cannulation technique are important factors in determining thrombus formation in assisted patients, as these will influence the intraventricular flow distribution in the LV. Several studies have been carried out to investigate the effect of inflow tip geometry [13-15] and inflow cannula size [6] on the intraventricular flow field. Using a numerical model, Liu et al. [13] concluded that the trumpet-tipped inflow cannula provides the best performance compared to the blunt, beveled and caged-tipped versions in providing smooth flow velocity distribution without backflow, low velocity flow or myocardial obstruction. Using a non-beating isolated heart, Bachman et al. [16] showed that compared to a beveled cannula, a flanged cannula reduces positional sensitivity to flow obstruction. In another study using computational fluid dynamics, Fraser et al. [6] compared the performance of three clinically available cannulae of varying diameters for pediatric circulatory support. They found that the risk of blood clotting at flow rates below 0.75 L/min was increased using a cannula with larger diameter. A recent article published by Yano et al. suggested that flow distribution in the LV with a catheter-type continuous flow blood pump was affected by the shape and positioning of the catheter tip inserted through the aortic valve [17].

In a recent study with 216 patients implanted with axial flow pumps, Schmid et al. [4] showed that patients with a long inflow cannula (34 mm tip length into the LV) exhibited better survival rates with lower incidence of cerebrovascular adverse events compared to those with a short inflow cannula (tip length of 24 mm). Laumen et al. [14] further investigated the effect of inflow cannula position on ventricular flow patterns to evaluate ventricular wash out using a pneumatically driven model of LV dilated cardiomyopathy. Results from particle image velocimetry showed that deeper cannulation led to a more distinctive vortex on the cannula tip with higher velocity along larger segments of the ventricular wall, improving LV outflow tract washout.

Although longer cannulae have been shown clinically to be beneficial to patients, the reasons remain unclear. Schmid et al. [4] hypothesized that the underlying causes may be related to the characteristics of the patient, surgical techniques and blood flow patterns. Therefore, the aim of the present study is to examine the correlation between LVAD cannula placement and the potential for thrombus formation using a fluid structure interaction (FSI) model of the passive LV incorporating an inflow cannula. Detailed analysis of factors which affect thrombosis, i.e. the intraventricular flow field (recirculation and stagnation regions), pressure, and shear stress along the LV wall was carried out using a numerical model. As compared to *in vitro* experimental studies, numerical simulations allow reproducible numerical experiments under repeatable identical conditions.

## Methodology

### Model description

**Figure 1:**
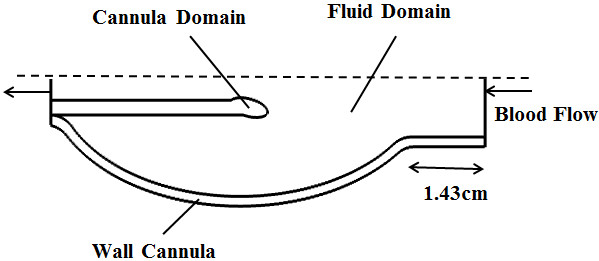
**Axisymmetric arrangement of cannula**, **fluid and LV wall domains.** The dashed line shows the axis of rotational symmetry and the arrow shows the direction of blood flow across the cavity during the filling phase.

**Figure 2:**
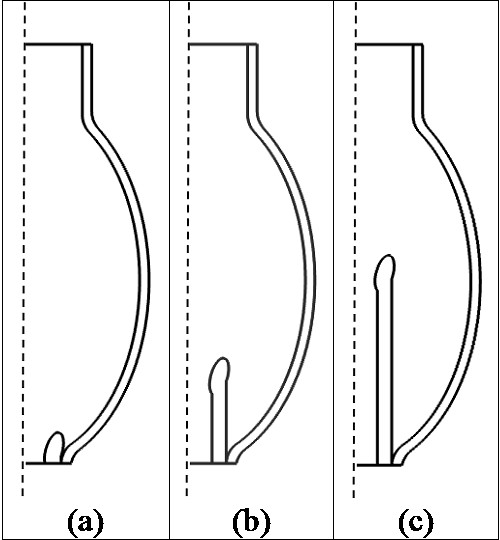
**The three different configurations of cannula placement used in the present study.** The three different configurations of cannula placement used in simulations. Three different configurations of cannula placement are used in the simulations: (**a**) near the apex; (**b**) one fourth of LV and (**c**) half LV.

As shown in Figure 1, the computational domain in our analysis includes an axisymmetric LV fluid domain, a silicone cannula inserted into the LV, and a hyperelastic LV wall. The LV is modeled using a prolate ellipsoid with a long axis of 8 cm, a short axis of 5 cm, and a wall thickness of 0.2 cm [18]. A 1.43 cm tube with a radius of 1.25 cm is connected to the upper end of the ellipse to represent the atrium. The diameter of cannula is set to 0.8 cm [19]. Three different configurations for cannula placement are simulated, as shown in Figure 2: (i) near the apex; (ii) one fourth of the LV length and (iii) half of the LV length. The trumpet tip design is used for the cannula following that suggested by many to reduce the risk of cannula obstruction [15,20].

The LV wall was modeled as a Mooney-Rivlin hyperelastic material with density 1200 kg.m^-3^[21] and strain energy density function, W_s_ as [22]:

(1) $$W_{s}=C_{10}\left(I_{1}-3\right)+C_{01}\left(I_{2}-3\right)+\frac{1}{2}k{\left(J_{\mathrm{el}}-1\right)}^{2}$$

Where *I*_1_ and *I*_2_ are the first and second invariants of the left isochoric Cauchy-Green deformation tensor, *J*_*el*_ is the elastic Jacobian, *C*_*i*,*j*_ are material parameters (*C*_*10*_ = 10 kPa, *C*_*01*_= 2 kPa), and *k* is the bulk modulus (100 kPa). The blood is assumed to be laminar, isotropic and non-Newtonian, with a density of 1008 kg.m^-3^. To model the non-Newtonian behavior of the blood, the Carreau-Yasuda model was used [23], whereby the relationship between the viscosity, μ, and the shear rate, $\dot{\gamma }$, is given bywhere μ_0_ = 13.15mPa∙s, *μ*_*∞*_ = 3mPa∙s, n = 0.4, and λ = 0.4. The cannula wall was modelled as a linear elastic silicone material with a density of 1110 kg.m^-3^, Young's modulus 3.6 MPa, and Poisson ratio of 0.49 [24].

(2) $$\mu =\mu_{\infty }+\left(\mu_{0}-\mu_{\infty }\right){\left[1+{\left(\lambda \dot{\gamma }\right)}^{2}\right]}^{\frac{n-1}{2}}$$

### Numerical scheme

The FSI module provided by the commercial software, COMSOL (COMSOL AB, Sweden), was chosen to solve the model. The module employed the arbitrary Lagrangian–Eulerian (ALE) method to solve the blood fluid flow formulated using an Eulerian description with LV wall deformation formulated using a Lagrangian description.

The fluid flow in the LV was described by the incompressible Navier Stokes equation (3) and the mass continuity equation (4):where *ρ* is the fluid density, u_f_ is the velocity vector of the fluid in a fixed coordinate system, u_m_ is the velocity vector of the fluid in a moving coordinate system, *p* is the pressure and I is the unit tensor.

(3) $$\rho \left(\frac{\partial u_{f}}{\partial t}+\left(u_{f}-u_{m}\right)\cdot \nabla u_{f}\right)=\nabla \cdot \left[-pI+\mu \left(\nabla u_{f}+{\left(\nabla u_{f}\right)}^{T}\right)\right]$$

(4) $$\rho \nabla \cdot u_{f}=0$$

Once the above equations are solved, the load exerted by the fluid on the LV wall (τ), i.e. the summation of the pressure and viscous stress, can be calculated using

(5) $$\tau =-pI+\mu \left(\nabla u_{f}+{\left(\nabla u_{f}\right)}^{T}\right)$$

This load from the fluid is then applied as a boundary condition to the LV wall at the fluid–solid interface boundary, according to

(6) τ·n=σ·n

where σ is the stress tensor in the LV wall and n refers to the interface unit normal. The calculated LV stress was then used to solve for the LV displacement, X, at every point in the wall:

(7) $$\rho \frac{\partial^{2}X}{\partial t^{2}}-\nabla \cdot \sigma =0$$

The resultant LV displacement was then used to calculate the fluid velocity, given that at the fluid-LV interface, u_w_ assuming the velocity of at the interface is equal to the velocity of the wall:

(8) $$u_{f}=u_{w}$$

Where

$$u_{w}=\frac{\partial X}{\partial t}$$

**Figure 3:**
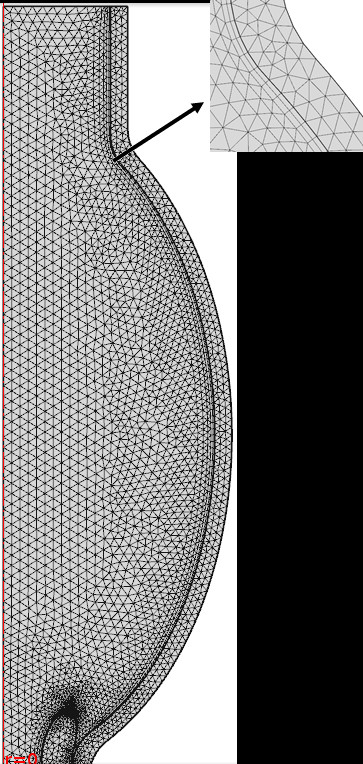
Mesh element detail at the interface boundary between the LV wall and blood fluid domains.

Steps (3)-(8) are iteratively repeated until the solution converges globally at each time point. This was carried out automatically in COMSOL using the FSI module, using the Parallel Direct Sparse Solver (PARDISO) solver in all simulations [25,26]. To examine the spatial convergence of the models, we compared simulation results using two types of mesh resolutions, the “fine” and “finer” resolutions provided by COMSOL. Both the fluid and solid LV domain were meshed with triangular and quadrilateral elements (Figure 3). The total numbers of mesh elements for two mesh resolutions are listed in Table 1.

**Table 1:** Total mesh elements for the various configurations of cannula placement at two mesh resolutions

**Cannula placement**	**“Fine” mesh**	**“Finer” mesh**
Near apex	5867 (mean element size = 0.10493 cm)	14027 (mean element size = 0.07648 cm)
One fourth LV	6896 (mean element size = 0.09910 cm)	15961 (mean element size = 0.07189 cm)
Half LV	7808 (mean element size = 0.09545 cm)	18706 ( mean element size = 0.06606 cm)

**Figure 4:**
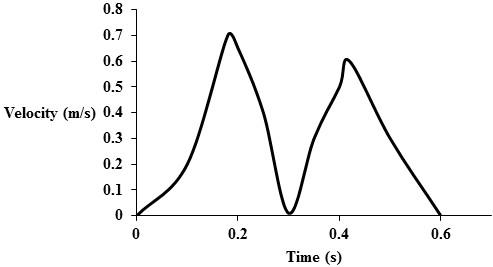
Transmitral velocity profile is applied at the inlet.

For the Navier–Stokes equation (3), a transmitral velocity profile adapted from [27] (as shown in Figure 4) was applied as a boundary condition at the inlet boundary to simulate LV filling. At the outlet boundary, a zero pressure boundary condition was applied.

## Results

### Spatial convergence of the model

To examine the spatial convergence of the model, we compared the maximum pump flow and average intraventricular pressure obtained using the “fine” and “finer” mesh options of Table 1. The maximum pump flow was defined as the maximum flow rate through the cannula outlet in the simulations. Resulting differences in maximum pump flow and average intraventricular pressure between the two mesh resolutions was 0.6% and 0.4% respectively, which we deemed to lie within an acceptable range. In order to reduce the computational time, the “fine” mesh setting was chosen for all subsequent simulations.

### Influence of cannula placement on vortex dynamics

**Figure 5:**
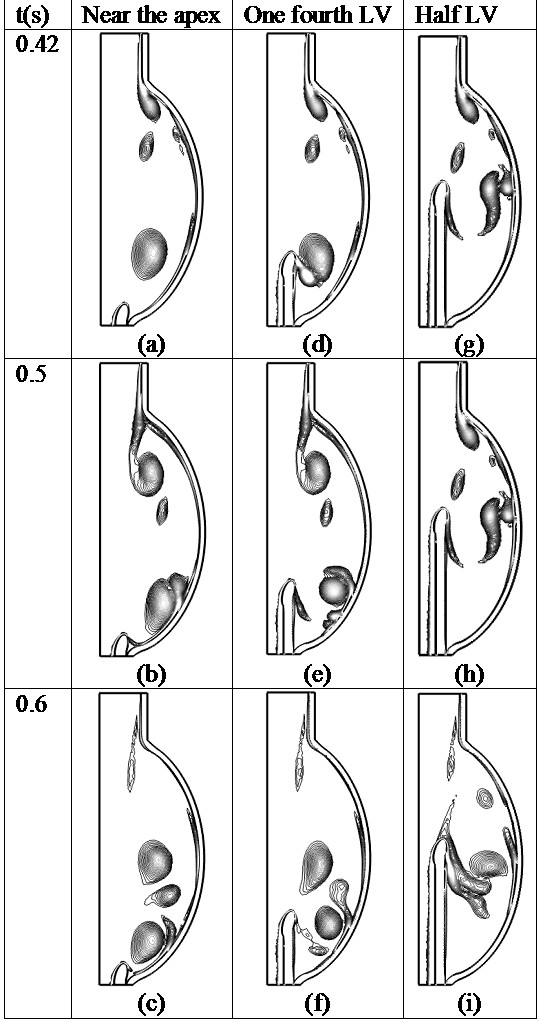
**Instantaneous vorticity scalar fields at different time points (t = 0.42, 0.5 and 0.6 s) showing isolevel contours plot (50 to 300, step = 10, unit = 1/s) for different cannula placements.** The vorticity scalar magnitude is shown, with direction perpendicular to the plane of the figures. (**a**) near the apex, at t = 0.42s; (**b**) near the apex, at t=0.5s; (**c**) near the apex, at t=0.6s; (**d**) one forth LV, at t = 0.42s; (**e**) one forth LV, at t=0.5s; (**f**) one forth LV, at t=0.6s; (**g**) half LV, at t = 0.42s; (**h**) half LV, at t=0.5s; (**i**) half LV, at t=0.6s.

In order to visualize the vorticity (∇ × **u**_**f**_) distribution under different cannula placements, the vorticity isolevel contour plot for all three placements are plotted in Figure 5 for various time points (t = 0.42, 0.5 and 0.6 s). Regardless of the difference in cannula placement, vortex formation patterns in the LV during the initial filling phase are similar for all cases. A vortex sheet is formed near the mitral annulus due to the incoming flow, which then develops into a primary vortex ring and travel downwards. Obvious changes in the vortex distributions are observed when the primary vortex ring comes into contact with the cannula. In the first scenario, when the cannula is inserted near the apex, the vortex ring is allowed to travel to the apical wall and interacts with the cannula to form a shear layer at the cannula tip (Figure 5a-c). When the cannula is inserted into one fourth of the LV, the primary vortex ring interacts earlier with the cannula wall (Figure 5d). The shear layer grows rapidly and induces an upward velocity which combines with the primary vortex ring to form a large vortex area at the cannula tip. At t = 0.5 s, the vortex ring detaches from the cannula tip and forms vortices near the apical wall of the LV (Figure 5e-f). When the cannula is inserted into half LV, the primary vortex ring interacts with the cannula tip before it reaches the LV apex (Figure 5g). Large vortices are observed near the mid-ventricular wall due to the interaction between the primary vortex ring and the ventricular wall. One important feature when the cannula is inserted into the half LV position is that a clear vortex shedding mechanism is observed at the outer surface of the cannula wall due to the interactions between the secondary incoming vortex, the primary vortex and the shear layer at the ventricular wall (Figure 5i).

**Figure 6:**
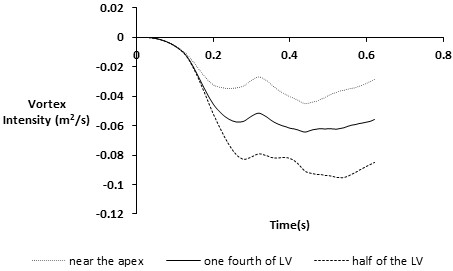
Time course of the average vortex intensity in the model under different cannula placements.

Figure 6 shows the temporal waveforms of average LV vortex intensity with different cannula placements. Negative values of vortex intensity represent clockwise rotation of the vortices. During the initial filling phase, the magnitude of the vortex intensity was similar in all cases due to similar vortex formation patterns. When the primary vortex ring came into contact with the cannula, the induction of shear layers and vortices at both the cannula tip and the ventricular wall led to a significant increase in vortex intensity. Compared to the case where the cannula was inserted near the apex and at one fourth of the LV, the half LV scenario exhibited the highest vortex intensity, which is four-fold and two-fold higher than the apex and one fourth LV placements respectively.

### Influence of cannula placement on velocity distribution

**Figure 7:**
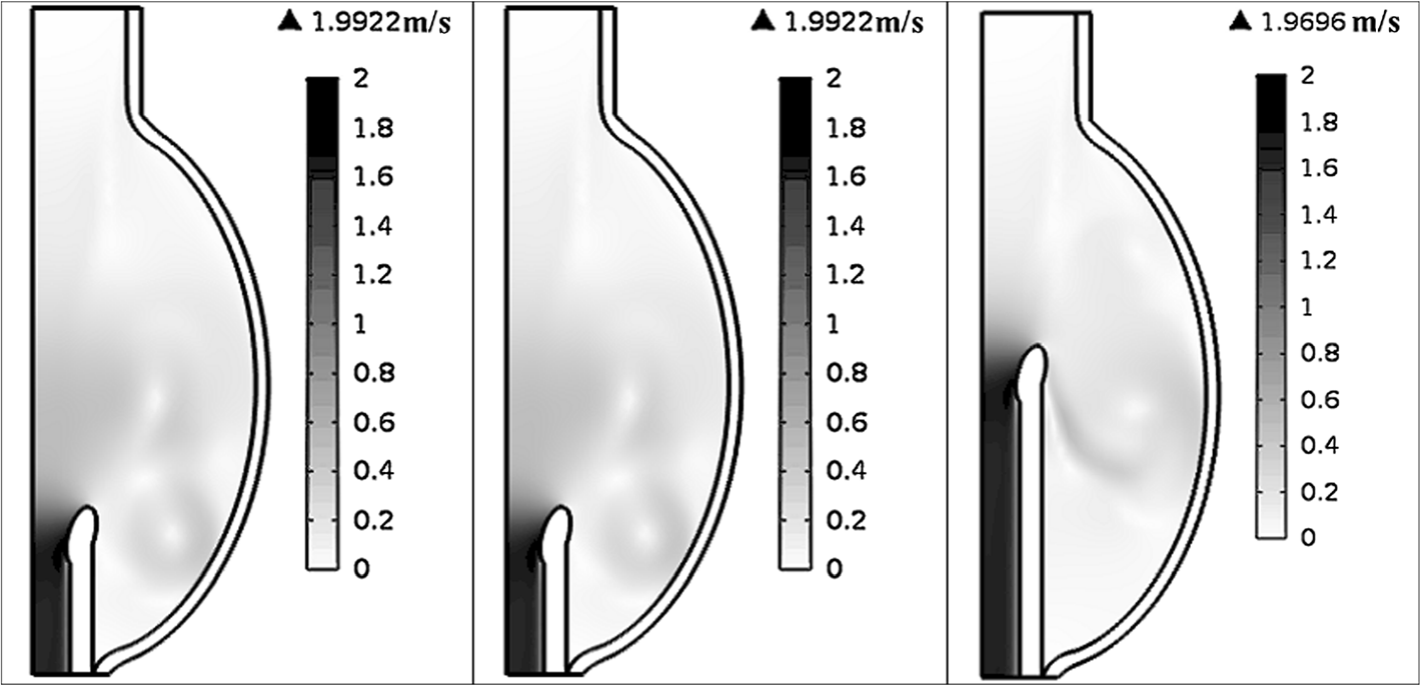
**Velocity magnitude spatial distribution in the model at t = 0.6 s under different cannula placements.**

**Figure 8:**
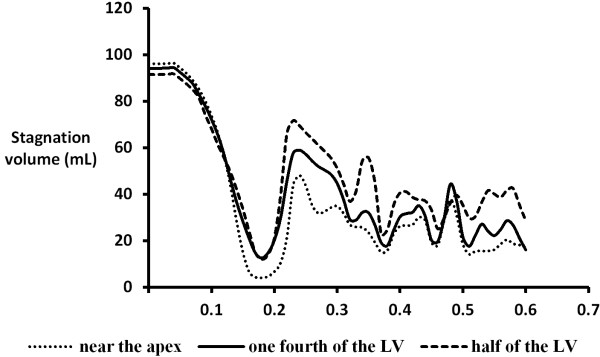
Time course of total stagnation volume under different cannula placements.

Figure 7 shows the spatial distribution of velocity at the end of filling (t = 0.6s). In all cases, higher velocities were found along the cannula, particularly near the inner wall of the cannula tip. Highest velocities were achieved when the cannula was inserted into one fourth of the LV (1.99 ms^-1^), followed by half LV (1.97 ms^-1^) and near the apex (1.81 ms^-1^). On the contrary, stagnation flow regions, defined as any regions with a velocity lower than 0.05 ms^-1^ (excluding the inlet which is governed by the velocity of the incoming flow), can be observed at the apical region (at all time points) and near the ventricular wall (different regions at different time points). Overall, the largest stagnation volume was found when the cannula was inserted half the LV (at all time points), where most stagnation occurs at the apical region of the LV. Insertion of the cannula into one fourth of the LV and near the apex yields similar stagnation volumes. At the end of filling, total stagnation volumes in the half LV, one fourth LV and near apex cannula placements was 28.5, 15.6 and 17.1 mL respectively, with the lowest velocity of 0.049 ms^-1^ found at the apical region in the half LV case. Time evolution of the total stagnation volume in the model under different cannula placements is shown in Figure 8.

### Influence of cannula placement on wall shear stress distribution

Shear stress along the inner wall of the LV was found to bebelow5 Pa in all cases at all time points. Generally, different cannula placements yielded similar shear stress distributions at the ventricular wall during the early filling phase (before the primary vortex ring comes into contact with the cannula wall). After t = 0.42s, changes in the vortex patterns and spatial velocity profiles produces higher shear stresses at the apical wall region when the cannula is inserted near the apex (up to 5 Pa near the apex). This is followed by the one fourth LV (up to 3.5 Pa) and half LV (up to 2.2 Pa) placement scenario. Compared to the shear stress at the ventricular wall, much higher shear stresses were found along the inner wall of the cannula, particularly at the cannula tip, due to a higher velocity and shear rate. Placement of the cannula at half LV yields the highest cannula wall shear stress, i.e. 28.3 Pa, followed by that for the one fourth LV scenario (28.0 Pa) and insertion near the apex (25.8 Pa). The difference in maximum shear stress among the three cannula placements was approximately 10%.

## Discussion

To date, detailed investigations into the patterns of intraventricular vortices as well as quantitative analysis of vortex dynamics, including the vortex core area, intensity and propagation velocity, has attracted wide interests [28]. It has been reported that cardiac disorders can lead to alterations in the vortex dynamics [29] and serve as a useful diagnostic tool. In the present study, highest vortex intensity was achieved when the cannula was inserted half-way into the LV. This was due to the interaction between the primary vortex ring and a secondary incoming one which induces large vortices and shear layers near the cannula and ventricular wall, particularly near the middle of the ventricle. Our results are consistent with the experimental findings of Laumen et al. [14], who observed a more distinctive vortex when the cannula was inserted deeper into the LV. Nevertheless, since their results were based on horizontal cut planes viewed from the top of the LV, it is difficult to determine the precise location of the vortices between the base and the apex. On the contrary, the result of this study showed that the lowest vortex intensity was obtained with the cannula near the apex, due to a lack of vortices in the upper half of the LV. As noted by Laumen et al. [14], incoming flow entering the ventricle is mostly sucked into the cannula without much interaction with the ventricular wall. High vortex intensity in the LV has been reported to be beneficial helping to improve wash out at the ventricular wall [14], reducing the risk of thrombus formation.

However, insertion of the cannula halfway into the LV produces thick shear layers and vortices near the cannula tip, which prevents the vortices from travelling to the apex. As a result, a small stagnation flow region is found at the apical region of the LV, which may promote the accumulation of platelets and consequently thrombus deposition at the apex. Our results agree with Antaki et al. [15], who suggested that deep cannula insertion could result in blood stagnation at the ventricular apex. On the other hand, total stagnation flow volume when the cannula is inserted at both the one fourth LV and apical positions is much lower.

The vortex shedding mechanism observed at the outer surface of the cannula (most obvious in the half LV case) may be related to the clinical findings of Miyake et al. [30], which indicated the formation of a LV thrombus adjacent to the inflow cannula in a patient supported with LVAD apical cannulation. It has been reported that the vortex shedding phenomena provides optimal mixing for platelet aggregation, increases the procoagulant surfaces needed for the coagulation reactions and disperses the clotting factors in the process [31]. This promotes the adhesion of platelets on the surface of the cannula.

High shear stress in blood recirculating devices has often been associated with platelet activation and hemolysis. Hemolysis requires a shear stress of more than 150 Pa [32], which was not found in our simulations. In a recent study, Wu et al. [33] reported that the appropriate threshold shear stress for platelet activation lies between 1.2 Pa (the physiological shear stress) and 10.5 Pa (significant platelet activation). Our results showed that all three cannula placement scenarios produced a shear stress exceeding 10 Pa (maximum of 28.3 Pa found at the cannula tip in the half LV case) at the inner wall of the cannula wall, which may result in significant platelet activation. On the contrary, lower shear stress was found at the ventricular wall, with a maximum of 5 Pa found at the apical region of the LV when the cannula was located near the apex. Along with the shear layer attaching to the cannula and the ventricular wall, this may induce a small amount of platelet activation.

Our results show that placement of the cannula into one fourth of the LV achieves the best performance in terms of reducing the risk of thrombus formation. Compared to cannula placement near the apex, higher vortex intensity is achieved in the one fourth placement where more interactions occur between the vortices and the ventricular wall (particularly in the upper half of the LV), thus increasing wash out of the platelets. Moreover, wall shear stress at the apex when the cannula is inserted into one fourth of the LV is almost 50% lower than that of the apex placement. On the other hand, the one fourth placement has a negligible amount of stagnation flow near the apical wall of the region, compared to the half LV case, which may increase the likelihood of platelet deposition. Our numerical results may explain the clinical findings of Schmid et al. [4], who showed that patients with a long inflow cannula (34 mm tip length into the LV) exhibited a lower incidence of cerebrovascular adverse events compared to those with a short inflow cannula (tip length of 24 mm). Referring to the figures of their article, the long inflow cannula corresponds to the one fourth LV scenario in the present study, while the short inflow cannula corresponds to the apex LV case.

### Limitations

In this study, an idealized geometry with smooth surface and isotropic material properties are used. In reality, the LV is a complex geometry with non-smooth endocardial surface and anisotropic material properties, which may result in localized stress distributions. In addition, the geometry has not been optimized for a congestive heart failure patient with remodeled myocardium, due to limited clinical and experimental observations of a remodeled myocardium in the presence of an LVAD. Furthermore, as pointed out by Baccani et al. [34], during a large part of the diastolic phase, the geometry and wall properties of the ventricle have a minor relevance in the ventricular flow. Secondly, a zero stress condition was applied to the cannula outlet in our simulations. Physiologically, the outlet pressure at the cannula depends on the impedance of the systemic circulatory system.

## Conclusion

We have presented a FSI model of the LV incorporating an LVAD outflow cannula, to study the effect of cannula placement on vortex dynamics, velocity distribution and wall shear stress. In all cases, vortex shedding was observed at the outer surface of the cannula, which may promote the adhesion of platelets on the cannula surface. Furthermore, shear stresses of more than 10 Pa were observed at the inner surface of the cannula wall, which may result in significant platelet activation. When the cannula is inserted near the apex, less interaction occurs between the vortices and the ventricular wall, resulting in lower vortex intensities compared to the one fourth and half LV cannula placements. When the cannula is inserted half the LV, the strong interaction between the shear layers at the cannula tip, the primary vortex ring and secondary vortices prevents the vortices from travelling to the apical region of the LV, resulting in a small stagnation flow region. In summary, our results show that placement of the cannula into one fourth of the LV achieves the best performance in terms of reducing the risk of thrombus formation. Future work will include modelling the contractile activity of the LV to simulate the whole cardiac cycle, as well as validating model results with*in vitro* and *in vivo* experiments.

## Competing interest

The authors declare that they have no competing interests.

## Authors’ contributions

CWO carried out the simulation of fluid structure interaction and drafted the manuscript. SD provided guidance in the simulation of fluid structure interaction and participated in the manuscript editing. BTC provided assistance in the simulation and participated in the manuscript checking. EL provided guidance in the simulation of fluid structure interaction and participated in the manuscript writing. AAA provided assistance in the simulation and participated in the manuscript editing. NAO provided assistance in the simulation and manuscript checking. SK provided assistance in the simulation submission progress and manuscript checking. NHL provided assistance in the simulation running and manuscript editing. All authors read and approved the final manuscript.
